# Autistic Behavior as Novel Clinical Finding in OFD1 Syndrome

**DOI:** 10.3390/genes14020327

**Published:** 2023-01-27

**Authors:** Sorina Mihaela Papuc, Alina Erbescu, Adelina Glangher, Ioana Streata, Anca-Lelia Riza, Magdalena Budisteanu, Aurora Arghir

**Affiliations:** 1 Medical Genetics Laboratory, Victor Babes National Institute of Pathology, 050096 Bucharest, Romania; 2 Psychiatry Research Laboratory, Prof. Dr. Alex. Obregia Clinical Hospital of Psychiatry, 041914 Bucharest, Romania; 3 Regional Centre of Medical Genetics Dolj, Emergency County Hospital Craiova, 200642 Craiova, Romania; 4 Laboratory of Human Genomics, University of Medicine and Pharmacy of Craiova, 200638 Craiova, Romania; 5 Department of Genetics, Faculty of Medicine, Titu Maiorescu University, 031593 Bucharest, Romania

**Keywords:** ciliopathy, autism, neuronal migration

## Abstract

Orofaciodigital syndrome I (OFD1–MIM #311200) is a rare ciliopathy characterized by facial dysmorphism, oral cavity, digit, and brain malformations, and cognitive deficits. OFD1 syndrome is an X-linked dominant disorder reported mostly in females. The gene responsible for this condition, OFD1 centriole and centriolar satellite protein (*OFD1*), is involved in primary cilia formation and several cilia-independent biological processes. The functional and structural integrity of the cilia impacts critical brain development processes, explaining the broad range of neurodevelopmental anomalies in ciliopathy patients. As several psychiatric conditions, such as autism spectrum disorders (ASD) and schizophrenia, are neurodevelopmental in nature, their connections with cilia roles are worth exploring. Moreover, several cilia genes have been associated with behavioral disorders, such as autism. We report on a three-year-old girl with a complex phenotype that includes oral malformations, severe speech delay, dysmorphic features, developmental delay, autism, and bilateral periventricular nodular heterotopia, presenting a de novo pathogenic variant in the *OFD1* gene. Furthermore, to the best of our knowledge, this is the first report of autistic behavior in a female patient with OFD1 syndrome. We propose that autistic behavior should be considered a potential feature of this syndrome and that active screening for early signs of autism might prove beneficial for OFD1 syndrome patients.

## 1. Introduction

Orofaciodigital syndrome I (OFD1-MIM #311200) is a rare, complex, and clinically variable disorder characterized by facial dysmorphism and malformations of the oral cavity (mouth, tongue, and teeth) and digits [1]. OFD1 syndrome is an X-linked dominant disorder diagnosed only in female patients due to male lethality. The first reports date back to the mid-twentieth century [2,3] and include families and sporadic cases with oral, facial, and digital anomalies and renal and brain malformations, which led to the delineation of a distinct clinical entity [2,3,4].

The genetic cause of OFD1 was discovered in 2001 by Ferrante et al. [5], who performed a variant analysis of genes located in the critical region Xp22 in a group of familial and sporadic OFD1 cases. All patients with the clinical presentation of OFD1 syndrome had mutations in the Chromosome X open reading frame 5 (*CXorf5)* gene, later referred to as the *OFD1* gene (MIM 300170). The variants in this gene were also reported in developmental conditions with an X-linked recessive pattern of inheritance, included in the OMIM database: Joubert syndrome 10 (JBS10, MIM #300804), Simpson-Golabi-Behmel syndrome, type 2 (SGBS2-MIM #300209), and Retinitis pigmentosa 23 (RP23, MIM #300424, provisional association). However, subsequent studies have found that hypomorphic variants in the phosphatidylinositol glycan class A (*PIGA*) gene are responsible for a spectrum of clinically diverse neurodevelopmental disorders, including SGBS2 [6]. Thus, it has been suggested that the *OFD1* gene may not be responsible for SBGS2 [7,8].

The *OFD1* is involved in primary cilia formation, left-right axis specification [9,10], regulation of the length and distal structure of centrioles [11], chromatin remodeling, and DNA repair [12]. The primary cilia are linked to multiple signaling pathways and play fundamental roles in the development of several organs, including the cerebral cortex. The functional and structural integrity of cilia impacts critical brain development processes, such as neurogenesis, neuronal migration, and neural circuitry establishment [13,14]. This explains the broad range of neurodevelopmental anomalies in ciliopathy patients [14]. Anomalies of the central nervous system are reported in approximately 50% of patients [15]. In addition, cognitive defects of variable severity are detected, as are multiple types of brain malformations [16,17]. The gray matter heterotopia is a recurrent feature of OFD1 syndrome, as reported in several patient cohorts [16,17,18,19,20,21]. The damaged variants in cilia genes lead to structural and functional alterations of the primary cilia resulting in neurodevelopmental disorders. Given that several psychiatric disorders, such as ASD and schizophrenia, are neurodevelopmental in nature, their connections with cilia roles are worth exploring.

Several cilia genes have been reported in association with behavioral problems, such as autism, although psychiatric disorders associated with cilia genes have not been in the focus of clinical or basic research until recently [22,23]. ASD represent a heterogeneous group of neurodevelopmental conditions characterized by a specific combination of social relationships and communication deficiencies, repetitive behaviors, and restricted interests, with onset in early childhood [24]. ASDs have long been recognized to have an important genetic basis [22,25,26], although other contributing factors, such as environmental factors may also be involved [22,27]. The first evidence of the genetic susceptibility to ASD was provided by family linkage analysis and twin studies [28,29,30]. Further on, the association of autism with rare monogenic disorders, such as tuberous sclerosis, Rett, Joubert, and Fragile X syndrome, provided additional support for the genetic etiology of ASD [25,26,31]. The extensive use of genomic technologies, such as next-generation sequencing and chromosomal microarrays, led to an extensive characterization of the genetic architecture of ASD and the identification of a wide variety of rare variants with major contributions to ASD [32,33,34,35,36,37]. Some of these variants directly disrupt cilia genes such as *AHI1*, *ARL13B*, *CEP290*, *INPP5E*, NPHP1, RPGRIP1L, and TMEM67 and thus, have major consequences on essential structural components of the cilia [23,38]. Others are localized in genes that indirectly interfere with cilia development and function, such as *FMR1*, *CNTNAP2*, and *FOXP1* [39,40]. In addition, certain copy number variants—for example, 16p11.2—known to alter the dosage of genes encompassed and possibly to influence the expression of other neighboring or distant genes, were also linked to cilia [41]. The primary cilia seem to play important roles in complex functions of the brain, and some of the patients with ciliopathies have neuropsychiatric disorders [1,38,42]. Furthermore, the knockdown of genes known as risk factors for neuropsychiatric phenotypes led to reduced ciliation [39]. Thus, interest in the underlying molecular mechanisms that link primary cilia and psychiatric disorders has been renewed in the last few years due to clinical and genetic evidence [43,44,45].

We report on a three-year-old girl with a complex phenotype that includes oral malformations, severe speech delay, dysmorphic features, developmental delay (DD), autism, and bilateral periventricular nodular heterotopia presenting a de novo pathogenic variant in the *OFD1* gene. To the best of our knowledge, this is the first report of autistic behavior in a patient with OFD1 syndrome.

## 2. Materials and Methods

The patient is a three-year-old girl with severe speech delay, DD, and autistic behavior. She was evaluated by general clinical, neurological, and psychiatric examinations; psychological evaluation (Portage test for development quotient and Autism Diagnostic Observation Schedule-ADOS-Module 2 for autistic behavior); electroencephalographic studies (EEG); and 3 Tesla brain magnetic resonance imaging (MRI). However, no ultrasound investigation was performed on our patient. Blood samples for biological tests and genetic investigations were drawn from the patient, her parents, and her older brother.

DNA was extracted from whole blood using a commercial kit (PureLink Genomic DNA Mini Kit, ThermoFisher Scientific, Waltham, MA, USA). The concentration and purity of the DNA samples were quantified using a NanoDrop 2000 spectrophotometer (ThermoFisher Scientific) and Qubit 2.0 Fluorometer (ThermoFisher Scientific). Genetic investigations included array-based comparative genomic hybridization (array-CGH) (Agilent Technologies, Santa Clara, CA, USA), Filamin A (*FLNA*) gene Sanger sequencing and whole exome sequencing (WES) performed in a trio (child and both parents).

An array-CGH experimental protocol using a 180K k oligonucleotide array (SurePrint G3 Human CGH Microarray Kit 180K, Agilent Technologies) was performed according to the manufacturer’s recommendations, starting from 1000 ng genomic DNA (gDNA). In brief, gDNA was digested with AluI and RsaI and subsequently fluorescently labeled using the SureTag DNA Labeling Kit (Agilent Technologies). Agilent Human Reference female DNA was used as a reference and labeled with cyanine 5 deoxyuridine triphosphate (Cy5-dUTP), while patient DNA was fluorescently labeled with Cy3-dUTP. After 24 h of hybridization followed by post-hybridization washes, the oligonucleotide slides were scanned using the Agilent SureScan Microarray Scanner System. Agilent CytoGenomics Software v5.2 (Agilent Technologies) was used for raw data extraction and data analysis. Data interpretation was performed as described elsewhere [46].

Additionally, Sanger sequencing of coding and splice-site regions of the *FLNA* gene was performed on an ABI 3500 Genetic Analyzer (Applied Biosystems, Foster City, CA, USA). The PCR products were generated using primers from Oda et al. [47]. The final reaction volume was 25µL and included for each primer pair: 0.1 μL Invitrogen Taq Polymerase Recombinant (5 U/μL, ThermoFisher Scientific), 2.5 μL reaction buffer 10X, 0.75 μL MgCl2 (50 mM), 0.5 μL dNTP (10 mM), 0.5 μL (10 μM) of forward and reversing primer, respectively, 50 ng gDNA, and nuclease free water. PCR amplification was performed under the following conditions: denaturation at 94 °C for 3 min, 35 cycles consisting of denaturation at 94 °C for 45 s, primer annealing at 56 °C for 45 s, elongation at 72 °C for 1 min, and a final elongation step at 72 °C for 5 min. Sanger sequencing reactions were performed using the BigDye Terminator v3.1 Cycling Sequencing Kit (Applied Biosystems) following the experimental protocol previously described [48].

Library preparation was performed using the Ampliseq Exome Panel for Illumina and the AmpliSeq Library PLUS for Illumina (Illumina, San Diego, CA, USA) starting from 100 ng gDNA according to the manufacturer’s protocol. After target amplification, the amplicons were partially digested using FuPa reagent, followed by ligation of indexes using DNA ligase and AmpliSeq CD-Indexes large volume (Illumina). The barcoded libraries were purified using AMPure XP beads (Beckman Coulter, CA, USA) and further enriched and normalized using the AmpliSeq Library Equalizer for Illumina (Illumina). The enriched libraries were denatured and sequenced on Illumina NextSeq 550Dx equipment and NextSeq 500/550 High Output Kit v2.5 (300 cycles) (Illumina). The paired-end sequences were aligned to the human reference genome build GRGh37/hg19 using NextGENe software v.2.4.2.3 (SoftGenetics, State College, PA, USA). Variants were called with the same software if present in at least 20% of reads with a good quality level and further annotated to dbSNP 153 and dbNFSP v.3.5, which include *in silico* prediction tools such as SIFT (http://sift.jcvi.org/, last accessed on 4 November 2022), PolyPhen2 (http://genetics.bwh.harvard.edu/pph2/, last accessed on 4 November 2022), LRT, MutationTaster (http://www.mutationtaster.org, last accessed on 4 November 2022), MutationAssessor, FATHMM, GERP, and CADD (http://cadd.gs.washington.edu, last accessed on 4 November 2022). A trio analysis was performed for the detection of de novo, compound heterozygous, and homozygous calls in coding and splice-site (including 20 intronic base pair) regions. The rare variants (with an overall minor allele frequency below 1%) were evaluated using VarSome [49], OMIM [1], and ClinVar (https://www.ncbi.nlm.nih.gov/clinvar/, last accessed on 4 November 2022) and categorized in accordance with American College of Medical Genetics recommendations [50].

This study was approved by the ethics committees of the institutions where it took place, namely, Prof. Dr. Alex. Obregia Clinical Hospital of Psychiatry, Bucharest, Romania (approval codes 32190/16.10.2019 and 33/26.11.2019) and Victor Babes National Institute of Pathology, Bucharest, Romania (approval codes 76/3.12.2019 and 68/14.09.2019). Written informed consent for participation in the study and for data publication was obtained from the parents of the patients before inclusion in the study.

## 3. Results

### 3.1. Clinical Case Presentation

A three-year-old girl was referred for neurogenetic evaluation in the context of severe speech delay and a history of tongue malformation. The patient is the second child of non-consanguineous healthy parents, born after an uneventful pregnancy and birth, with a birth weight of 3250 g, a birth length of 50 cm, an Apgar score of 9, and good postnatal adaptation. She has an older brother with Asperger syndrome and 22q11.2 duplication syndrome (MIM #608363). In the first days of life, she was diagnosed with tongue malformation (bifid tongue and oral choristoma), which was operated on at the age of 1 year and 10 months. Her psychomotor development was normal in the first year of life (she walked at 12 months and spoke her first words at 11 months). The parents declared that at the age of 22 months, she experienced cognitive regression, losing all meaningful words and interest in interaction with other children. The parents associated this regression with the surgical intervention for her tongue tumor, but there were no medical records regarding the psychomotor development of the girl within the interval of 12–22 months of age. At two years of age, she was evaluated in the department of child psychiatry, and a diagnosis of ASD was established. She began cognitive-behavioral therapy but without significant progress, especially concerning language. Her clinical evaluation, at the age of three years, revealed normal growth parameters (weight 14 kg, Pc 53; length 91 cm, Pc 17; occipital-frontal circumference 47 cm, Pc 15) and dysmorphic features: deep-set eyes, anteverted nostrils, a high arched palate, a bifid tongue, missing central inferior incisors, and microretrognathia. The neurological examination showed severe speech delay (she says only “mama” and performs no orders) and autistic behavior (poor eye contact, stereotypic movements, and difficulties in social interaction and adaptation to new situations). The parents denied any seizures or other paroxysmal events. The EEG was normal. No digital malformations or skin anomalies were noted on the clinical examination of our patient. All biological blood tests were in the normal range. Her psychological evaluation revealed a development quotient of 50 (moderate DD) and an ADOS score of 20 (corresponding to moderate ASD).

### 3.2. Brain MRI Results

**Figure 1:**
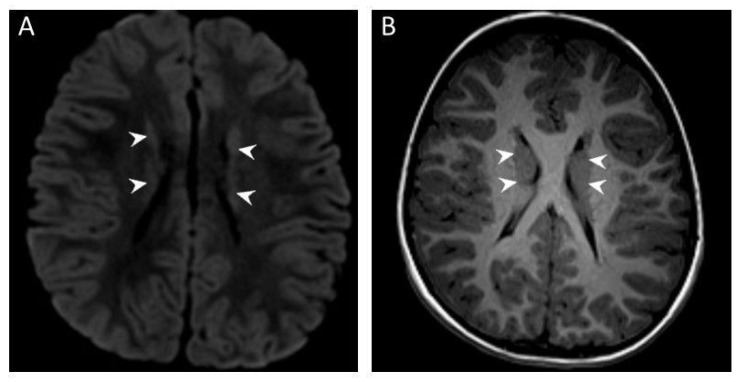
Brain MRI in our patient showing bilateral periventricular nodular heterotopia (arrowheads indicate ectopic gray matter nodules): T2 FLAIR (**A**) and T1 (**B**) axial brain MRI image.

The patient’s 3 Tesla brain MRI showed bilateral periventricular nodular heterotopia detected on both T2/T2 FLAIR- and T1-weighted images, with no other structural defect observed (Figure 1A,B).

### 3.3. Genetic Results

Array-CGH and *FLNA* variant screening did not reveal any defects with pathogenic or unknown significance.

WES reached an average sequencing coverage of 263-fold, with > 96% of the region of interest covered at least 20-fold. The analysis of WES data revealed a de novo variant in the *OFD1* gene (NM_003611.2:c.260A>G; NP_003602.1:p.Thy87Cys). This rare variant (rs312262818) was previously reported as pathogenic; functional predictions were deleterious for all computational methods used (CADD, PolyPhen2, LRT, Mutation Taster, SIFT, FATHMM, and PROVEAN). No other variants with pathogenic or unknown significance were detected in our patient.

## 4. Discussion

We report on a three-year-old female patient who presented the characteristic clinical features of OFD1 syndrome, oral and brain malformations, dysmorphic facial features, and a cognitive deficit. She also presented with severe speech delay and a previously unreported clinical trait, autistic behavior.

The OFD1 syndrome is a rare genetic condition with a dominant X-linked inheritance pattern that affects mainly females, with most males dying before birth. In addition, OFD1 syndrome has a wide range of clinical problems affecting many organs and systems. The most common clinical features of this syndrome include dysmorphic facial features (hypertelorism, hyponastic alae nasi, cleft upper lip, micrognathia), oral anomalies (tongue abnormalities—bifid or lobulated tongue, hamartoma; cleft palate, dental anomalies—missing or extra teeth, hypodontia; accessory gingival frenulum), abnormalities of the digits (syndactyly, brachydactyly, clinodactyly of the fifth finger), brain malformations (corpus callosum agenesis, cerebellar hypoplasia, heterotopia, intracerebral cysts), kidney anomalies (polycystic kidney disease) and DD/intellectual disability (ID). Other rare clinical findings may include pancreas, liver, and heart diseases, and skin and hair abnormalities [1].

Clinical variability is exceptionally high in OFD1 female patients, with variable expressivity observed even within affected individuals from the same family [4,16,51]. This variability refers to both the presence or absence of some clinical features and the severity of these features. In a study on 30 families with OFD1 syndrome, Bisschoff et al. found oral anomalies as the most constant features, present in all cases, while facial and digital anomalies were absent in some patients [16]. Other less often reported features included abnormal ears, decreased hearing acuity, congenital heart disease, upper airway infections, fibrocystic liver disease, and pancreas disease [16].

Only a few live-born male patients with *OFD1* gene mutations were reported until now. As observed in female patients, males’ phenotypes were also variable and included cleft palate and other oral anomalies, dysmorphic facial features, postaxial polydactyly, hydrocephalus, corpus callosum agenesis, and renal and heart malformations [52,53,54,55,56].

Alterations of the *OFD1* gene are considered the cause of this condition. The *OFD1* gene encodes a pleiotropic protein with partially understood biochemical functions [57]. *OFD1* is widely expressed in human adult tissues, such as the brain, pancreas, kidney, heart, skeletal muscle, liver, lung, and placenta [58]. Animal model studies have revealed that the Ofd1 protein is expressed during different stages of craniofacial structures and nervous system embryonic development [5,59]. OFD1 protein localizes to the centrosome, pericentriolar satellites, and basal body of the primary cilia [60,61], as well as in the nucleus [10].

The *OFD1* gene presents an *N*-terminal Lis1 homology (LisH) domain, five α helical coiled coil domains (α-CC), and an LC3 interacting region (LIR) domain recently described [10,62]. The α-CC is predicted to be involved in subunit oligomerization, and it has been demonstrated that OFD1 is able to self-associate through this region [10]. The LisH domain has a putative role in protein–protein interactions, stability, and/or localization of the protein and also may be involved in regulation of microtubule dynamics [63]. The LIR domain is located at the C-terminus of the protein in an unstructured region and has been shown to be involved in the regulation of autophagosome biogenesis [62]. Apart from its role in primary cilia formation and left-right axis specification [9,10], OFD1 is involved in the regulation of centriolar length [11], chromatin remodeling, DNA repair [12], cell cycle progression [64], and autophagy [62,65,66].

The mutational spectrum of the *OFD1* gene varies from single nucleotide changes (missense and truncating variants) to entire gene deletions, as recently reviewed by Pezzella et al. [8]. In total, 184 pathogenic or likely pathogenic variants were centralized from all published studies, of which 155 were reported in patients with the clinical presentation of OFD1 syndrome. The most frequently reported variants were point mutations (93%), with the highest prevalence of frameshift variants; the rest of 7% are represented by intragenic or whole *OFD1* gene deletions [8]. So far, no robust correlations have been observed between the variant type and the clinical description of OFD1 syndrome; however, the distribution of the vast majority of variants detected in this condition spans the first 17 of the 23 exons. The pathogenic variants beyond exon 17 are prevalent in male patients with recessive *OFD1* developmental disorders, including JBTS10, RP23, SGBS2, and other unclassified syndromes. The structural analysis of OFD1 protein revealed that most of the critical domains are coded by the first 17 exons, which explains the distribution of the pathogenic variants observed in *OFD1*-related disorders across the *OFD1* gene [67]. In OFD1 syndrome, several genotype-phenotype correlations were drawn for the clinical features of this condition, such as the association of alterations in exons 3, 8, 9, 13, and 16 with intellectual disability and cleft lip or palate [5,51].

The *OFD1* gene is localized on the Xp22.2 chromosome, thus X-inactivation may play a role in intra- and interfamilial clinical variability. Although *OFD1* is apparently not subjected to X-inactivation [58], the expression of the *OFD1* allele from the inactive X chromosome is decreased [68]. In addition, skewed inactivation was observed in various groups of OFD1 patients, suggesting that this phenomenon may play a role in the clinical variability [51,69]. However, Bisschoff et al. found no correlation between phenotypic severity and X-inactivation pattern, proposing that other modifying factors may contribute to the clinical variability of OFD1 syndrome [16].

The variant identified in our patient is localized in exon 3 (p.Thy87Cys) and was previously reported as pathogenic in three female fetuses with OFD1 syndrome features [21,70]. A phenotypic comparison with the cases harboring the same variant is hampered by the difference in developmental stage, thus a wider comparison with OFD1 syndrome patients was performed.

Our patient’s clinical presentation overlaps with the characteristic phenotype of OFD1 syndrome. The face and oral cavity anomalies are common features in this condition and include a cleft palate, a lobulated or bifid tongue, tongue hamartomas, a malformed oral frenula, and abnormal dentition, most of which are also present in our patient. The abnormalities of the digits, such as brachydactyly, syndactyly, clinodactyly of the fifth finger, polydactyly, and a duplicated hallux, are reported in 45% of patients with OFD1 syndrome (https://www.omim.org/entry/311200, accessed on 4 November 2022). Our patient had no digital malformations detected upon careful examination of the hands and feet. The biochemical investigation did not indicate any organ dysfunction, such as kidney, liver, or pancreas. However, due to the high risk of polycystic disease in OFD1 syndrome, the patient should be periodically monitored by abdominal ultrasound.

In addition, brain malformations are reported in about half of subjects with OFD1 syndrome and include, in most cases, corpus callosum anomalies (about 75–82%) [16,17], single or multiple epithelial or arachnoid cysts, but also abnormal gyration (14.3%), and heterotopia of the gray matter (~20% cortical malformation) [16,17]. Our patient also presented with a brain malformation, bilateral periventricular nodular heterotopia. Furthermore, a systematic clinical and radiologic assessment of complex central nervous system anomalies has been suggested early on for OFD1 syndrome patients [71]. However, cognitive deficits are reported in approximately half of these patients, varying from mild to severe [16]. Our patient had a moderate developmental delay, which aligns with the clinical data in the literature. Regarding our patient’s speech problems, no comparison with other patients was possible as there are no published data regarding the severity of this trait.

Our patient has ASD, and to the best of our knowledge, this is the first report of autistic behavior in a female patient with OFD1 syndrome. Other psychiatric problems, such as major depression [1], attention deficit hyperactivity disorder, and bipolar disorder [17], were rarely reported previously. A differential diagnosis between neurodevelopmental disorders such as intellectual disability and ASD can be complex because these disorders have some overlapping symptoms, especially concerning communication (e.g., speech delay and difficulties understanding orders). However, stereotyped movements and difficulties in social interactions are characteristics of ASD. Additionally, the psychological tests specific to ASD (ADOS and Autism Diagnostic Interview-Revised, ADI-R) are very useful in confirming autistic behavior. The association of autism with damaging variants in the *OFD1* gene was previously described only in a few male patients (Table 1). Two splice site variants and two missense variants were reported in these patients, affecting exons 11, 13, 16, and 17, respectively [56,71,72].

**Table 1:** Summary of previously reported ASD male patients carrying deleterious variants in *OFD1* gene.

Study	Diagnosis (Patient Cohort)	Inheritance	Genomic Position (hg19)	Variant Type (NM_003611.2)	Effect (NP_003602.1)	Affected Exons
Krumm, 2015 [72]	ASD (SSC collection)	De novo	chrX:13774696	c.1222-1G>T	Disruption of splice acceptor site of intron 12	Exon 13
Li, 2017 [73]	ASD (ASC/SSC collection)	Inherited	chrX:13771497	c.1066G>C	p.Glu356Gln	Exon 11
Sakakibara 2018 [56]	ASD (own cohort)	Inherited	chrX:13778441	c.2260+2T>G	Disruption of splice donor site of intron 16	Exons 16 and 17
Tran, 2020 [74]	ASD (own cohort)	Inherited	chrX:13778788	c.2209A>G (rs778936071)	p.Thr737Ala	Exon 16

ASD—autism spectrum disorders; SSC—Simon Simplex Collection; ASC—Autism Sequencing Consortia.

Although rare, male patients with pathogenic *OFD1* variants surviving beyond the neonatal period have been reported with a broad spectrum of ciliopathy clinical presentations, varying from simple conditions such as RP and primary ciliary dyskinesia to complex disorders involving multiple organ dysfunctions (JBST10) [75,76,77,78,79]. Given the wide clinical variability observed both in female and male patients with *OFD1* pathogenic variants, adding new cases may contribute to the expansion and refinement of the phenotypic spectrum.

## 5. Conclusions

The OFD1 syndrome is a complex genetic disorder that mainly includes dysmorphic facial features, oral and digital abnormalities, and different types of brain malformations. Our patient’s clinical presentation overlaps with the characteristic phenotype of OFD1 syndrome while presenting autism, a clinical feature unreported to date in this condition. Given the broad spectrum of clinical presentations reported in OFD1 syndrome, we propose that autistic behavior should be considered a potential feature of this syndrome and that active screening for early signs of autism may prove beneficial for OFD1 syndrome patients.

## Data Availability

The main data generated and analyzed in our study are included in this article.
